# Viability of an Early Sleep Intervention to Mitigate Poor Sleep and Improve Well-being in the COVID-19 Pandemic: Protocol for a Feasibility Randomized Controlled Trial

**DOI:** 10.2196/34409

**Published:** 2022-03-14

**Authors:** Kathleen Patricia O'Hora, Raquel A Osorno, Dena Sadeghi-Bahmani, Mateo Lopez, Allison Morehouse, Jane P Kim, Rachel Manber, Andrea N Goldstein-Piekarski

**Affiliations:** 1 Psychiatry and Behavioral Sciences Stanford University Stanford, CA United States; 2 Department of Psychology Stanford University Stanford, CA United States; 3 Sierra-Pacific Mental Illness Research, Education, and Clinical Center (MIRECC) Veterans Affairs Palo Alto Health Care System Palo Alto, CA United States

**Keywords:** insomnia, COVID-19, pandemic, telehealth, cognitive behavioral therapy, CBT-I, sleep, depression, well-being, telemedicine, impact, mental health, therapy

## Abstract

**Background:**

The COVID-19 pandemic has led to drastic increases in the prevalence and severity of insomnia symptoms. These increases in insomnia complaints have been paralleled by significant decreases in well-being, including increased symptoms of depression, anxiety, and suicidality and decreased quality of life. However, the efficacy and impact of early treatment of insomnia symptoms on future sleep and well-being remain unknown.

**Objective:**

Here, we present the framework and protocol for a novel feasibility, pilot study that aims to investigate whether a brief telehealth insomnia intervention targeting new insomnia that developed during the pandemic prevents deterioration of well-being, including symptoms of insomnia, depression, anxiety, suicidality, and quality of life.

**Methods:**

The protocol details a 2-arm randomized controlled feasibility trial to investigate the efficacy of a brief, telehealth-delivered, early treatment of insomnia and evaluate its potential to prevent deterioration of well-being. Participants with clinically significant insomnia symptoms that began during the pandemic were randomized to either a treatment group or a 28-week waitlist control group. Treatment consists of 4 telehealth sessions of cognitive behavioral therapy for insomnia (CBT-I) delivered over 5 weeks. All participants will complete assessments of insomnia symptom severity, well-being, and daily habits checklist at baseline (week 0) and at weeks 1-6, 12, 28, and 56.

**Results:**

The trial began enrollment on June 3, 2020 and closed enrollment on June 17, 2021. As of October 2021, 49 participants had been randomized to either immediate treatment or a 28-week waitlist; 23 participants were still active in the protocol.

**Conclusions:**

To our knowledge, this protocol would represent the first study to test an early sleep intervention for improving insomnia that emerged during the COVID-19 pandemic. The findings of this feasibility study could provide information about the utility of CBT-I for symptoms that emerge in the context of other stressors before they develop a chronic course and deepen understanding of the relationship between sleep and well-being.

**Trial Registration:**

ClinicalTrials.gov NCT04409743; https://clinicaltrials.gov/ct2/show/NCT04409743

**International Registered Report Identifier (IRRID):**

DERR1-10.2196/34409

## Introduction

### Background

The COVID-19 pandemic and resulting mass home confinement have led to a significant increase in insomnia complaints [1-3]. Recent studies from around the world report the prevalence of moderate to severe insomnia symptoms during the pandemic to range from 17.4% to 33.7% [4-6], compared with 10% to 15% prepandemic [7,8]. The prevalence rate is even higher among health care workers, individuals with medical comorbidities, and individuals in close contact with the virus [9,10]. Many of these insomnia symptoms may have developed as a direct result of the pandemic and home confinement [11]. For example, increases in sleep disturbances were directly influenced by the increase in COVID-19–related deaths, supporting the relationship between pandemic severity and insomnia severity [12]. Additionally, the increase in insomnia complaints may also be in part due to the exacerbation of several established risk factors for insomnia emerging from the COVID-19 pandemic including increased loneliness, perceived stress, and screen time, together with decreased social connection and physical activity [13-16].

The observed increases in insomnia complaints are paralleled by significant decreases in well-being, including increased depression, anxiety, and suicidality and decreased quality of life [17-21]. Critically, prior research in a nonpandemic environment found that insomnia is not only highly comorbid with depression and anxiety but also a strong predictive factor in their development and prognosis [22,23]. Given these findings, it is possible that the increase in insomnia complaints following the pandemic may be directly contributing to the increased prevalence of depression and anxiety during the pandemic [17,18]. Thus, treating insomnia symptoms early may be one approach to improve well-being and prevent future depression during the COVID-19 pandemic.

Cognitive behavioral therapy for insomnia (CBT-I) is the gold standard, first-line, nonpharmacological treatment for chronic insomnia recommended by the American College of Physicians [24]. It has been proven safe and effective in adults across the lifespan, in individual [25] and group formats [26] and when delivered in person or via telehealth [27]. A recently published article [28] from the European Academy for CBT-I provided evidence supporting the efficacy of CBT-I to treat sudden-onset insomnia and the validity of telehealth-delivered CBT-I. There is evidence that CBT-I not only reduces sleep complaints in those with chronic insomnia but can also reduce depression, anxiety, and suicidality and improve quality of life [29-33]. There is further evidence demonstrating that the resulting improvements in insomnia symptoms mediate the changes in depression symptoms but not the reverse [34]. Although the literature on this is limited, taken together, this evidence suggests that providing a brief, telehealth-delivered CBT-I to individuals with newly developed, pandemic-onset insomnia complaints may not only improve sleep but also improve well-being. However, to our knowledge, there are no studies investigating whether CBT-I would be an effective early treatment for insomnia arising from a highly disruptive and stressful event, such as a global pandemic, or whether intervening early in insomnia symptom onset could help mitigate other negative mental health outcomes. Additionally, it remains unknown whether common risk factors (eg, loneliness, perceived stress, and screen time) for insomnia and poor well-being that have been exacerbated by the pandemic may impact the effectiveness of an early intervention for insomnia symptoms.

Typically, individuals with sleep disturbance do not seek treatment unless their condition develops into chronic insomnia. This delay in seeking insomnia treatment makes parsing temporal, mechanistic relationships between insomnia and well-being nearly impossible. However, lifestyle changes and stress associated with the COVID-19 pandemic created large-scale disturbances in psychological well-being and sleep. These circumstances provided a novel opportunity to study the relationship between sleep and well-being by deploying an early sleep intervention to treat insomnia symptoms that have not yet developed into chronic insomnia. These circumstances have thus provided a unique opportunity through a pilot study to respond to a public health crisis and explore the temporal interrelationship between new sleep disturbances and deterioration in well-being as well as to assess whether intervening early in sleep disturbances is enough to alter these trajectories. The findings of the pilot study, the protocol of which is detailed in this paper, will be integral for guiding larger-scale trials in nonpandemic settings.

### Objectives

Our feasibility, pilot study investigates the viability of an early treatment for insomnia symptoms to treat insomnia symptoms arising during the COVID-19 pandemic and determine pandemic-related risk factors for worsening well-being and sleep outcomes. We also assess the impact of the intervention on insomnia severity and well-being across 28 weeks.

We will accomplish these objectives by conducting a waitlist-controlled trial across 28 weeks to address 3 aims.

Aim 1 is to determine whether a brief, telehealth CBT-I reduces insomnia symptoms arising during the COVID-19 pandemic. We hypothesize that CBT-I will lead to improvements in insomnia severity, as measured by the Insomnia Severity Index (ISI) [35,36], across 28 weeks. We also hypothesize that fewer participants in the CBT-I group will meet the Diagnostic and Statistical Manual of Mental Disorders 5th Edition (DSM-5) criteria for insomnia disorder, compared with the waitlist control group at weeks 12 and 28.

Aim 2 is to determine whether brief, telehealth CBT-I mitigates negative mental health outcomes arising during the COVID-19 pandemic. We hypothesize that, compared with the waitlist control group, the CBT-I group will have an improved trajectory of well-being across 28 weeks and will have better well-being at weeks 7, 12, and 28. Additionally, we hypothesize improvements in insomnia symptoms will mediate improvements in well-being from baseline to weeks 6, 12, and 28.

Aim 3 is to determine whether risk factors for insomnia that might be aggravated during the COVID-19 pandemic predict worse insomnia and negative mental health outcomes at follow-up. We hypothesize that self-reported high levels of social isolation, perceived stress, sleep reactivity, and screen time and low physical activity at baseline will predict worse long-term outcomes at 12 and 28 weeks across both study groups.

## Methods

### Trial Design

#### Overall Design

**Figure 1:**
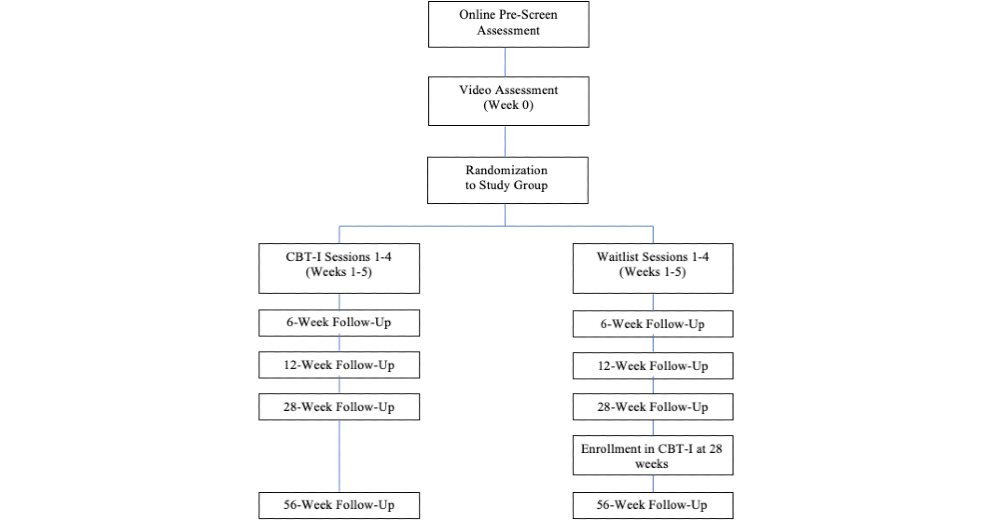
Study flow for each treatment group from prescreening through the 56-week follow-up, with primary outcome time points occurring at weeks 6, 12, and 28. CBT-I: cognitive behavioral therapy for insomnia.

We designed a 2-arm randomized controlled feasibility trial to investigate the efficacy of an early, brief, telehealth-delivered insomnia treatment to prevent adverse sleep and well-being outcomes. Participants with clinically significant insomnia symptoms (current ISI total score ≥10) that began during the pandemic were randomized to either a treatment group or a waitlist control group. Treatment consists of 4 telehealth sessions of CBT-I delivered over 5 weeks. Participants in the waitlist control group do not receive any study interventions during the 28-week primary assessment period. All participants complete assessments of insomnia symptom severity, depressive symptom severity, anxiety symptom severity, quality of life, and pandemic-related risk factors at baseline (week 0), weeks 1-6, week 12, week 28, and week 56 (Figure 1). Primary outcomes focus on weeks 6, 12, and 28.

**Figure 2:**
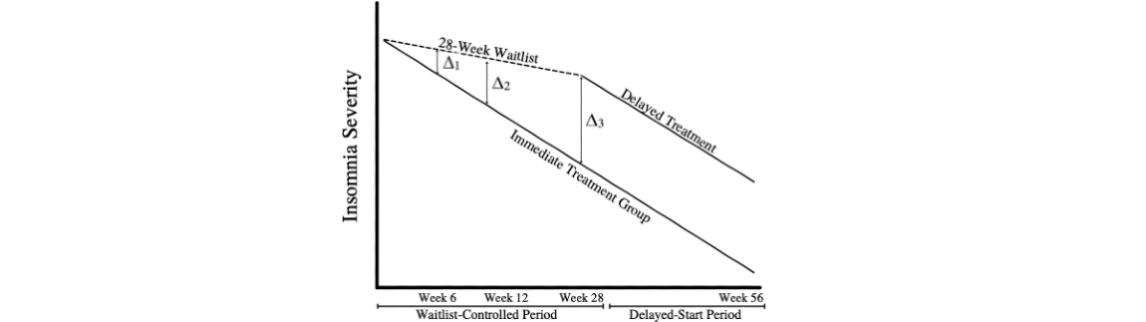
Study design in which the period between week 0 to week 28 is the waitlist-controlled period in which the immediate treatment group (cognitive behavioral therapy for insomnia [CBT-I]) can be directly compared with the waitlist control group (no CBT-I). At 28 weeks, participants in the waitlist group begin therapy and become the delayed treatment group.

This study design creates 2 study phases: a 28-week waitlist-controlled (primary assessment) period, which allows the assessment of the therapy compared with a treatment-naive group and a delayed-start (secondary assessment) period, during which participants originally assigned to the waitlist control group receive the study therapy and participants assigned to the treatment group no longer receive study treatment (Figure 2). This allows assessment of long-term changes in sleep and well-being between individuals who underwent an early behavioral intervention for insomnia and those who did not. Our innovative approach will help elucidate mechanistic pathways between sleep and well-being as well as provide a needed clinical response to the COVID-19 pandemic and resulting mental health crisis.

#### Randomization

Participants were randomized into either CBT-I or a 28-week waitlist control condition using stratification by biological sex at birth (male, female) and baseline insomnia severity (ISI ≥10, ISI <10) in a 1:1 ratio. Participants who dropped out of the study after randomization, but before the week 1 visit, were replaced in the randomization matrix. Our sample size estimates (see the Power Calculation section) accounted for this replacement.

#### Power Calculation

Due to the anticipated difficulties in recruitment that will likely arise from the complexities of running a study during a pandemic and the time-sensitive nature of implementing an early sleep intervention relative to both the start of the pandemic and insomnia symptoms, we view this trial as a feasibility study. Therefore, for this feasibility trial, we aimed to recruit a total of 50 subjects, which would result in 25 subjects in each study group. With this sample size, our power calculations were derived using the 2-group *t* test of equal means based on the difference between CBT-I and waitlist control group at 28 weeks, at a 5% level of significance. With 25 per group, the study is 80% powered to detect a large effect size (Cohen d=0.80).

#### Intervention

CBT-I is a comprehensive, multimodal approach that addresses maladaptive cognitions and behaviors that contribute to and maintain sleep difficulties. Treatment consists of education about the 2-process model of sleep (the homeostatic and circadian processes [37]) and their interaction with hyperarousal. Behavioral components of treatment include time-in-bed restriction (also known as sleep restriction [38]), stimulus control [39], and relaxation techniques. The cognitive component of treatment includes identification and modification of maladaptive beliefs and thought patterns about sleep to reduce sleep-related anxiety.

The study treatment protocol was adapted from Edinger’s 4-session, open-source CBT-I manual [40]. Treatment was “front-loaded,” in that Session 1 includes sleep education, stimulus control, and an initial time-in-bed restriction, based on sleep diary data collected in the 1 to 2 weeks prior to the first session. Sessions 2 through 4 are dedicated to adjusting time-in-bed based on ongoing sleep diary data, addressing treatment adherence issues, cognitive therapy, and relaxation skills. Session 4 also includes information about relapse prevention. Treatment is administered by a licensed psychologist or a doctorate-level graduate student trained and supervised by a licensed psychologist. The treatment is outlined in Table 1.

**Table 1:** Session by session outline of brief, telehealth cognitive behavioral therapy for insomnia (CBT-I).

Week	Session	Time	Content
1	1	60 minutes	Review the sleep log and answers provided on the brief sleep assessment. Educate about sleep and basic sleep hygiene instructions. Introduce two-process model of sleep (circadian rhythm and sleep drive) and interfering role of arousal. Determine standard wake time and initial time in bed (TIB) prescription. Provide stimulus control instructions. Answer questions and address concerns. Assign homework.
2	2	30-45 minutes	Review sleep log and adjust TIB prescriptions. Encourage/reinforce adherence. Identify/troubleshoot participant’s problems in adhering to recommended changes in sleep behaviors. Address sleep effort and sleep-related anxiety. Review role of arousal and teach relaxation technique. Answer questions and address concerns. Assign homework.
3 or 4	3	30-45 minutes	Review sleep log and adjust TIB prescriptions. Encourage/reinforce adherence. Identify and troubleshoot the participant’s problems in adhering to prescribed interventions (TIB, relaxation). Address sleep effort and sleep-related anxiety. Answer questions and address concerns. Assign homework.
4 or 5	4	30-45 minutes	Review sleep log and adjust TIB prescriptions. Encourage/reinforce adherence. Identify and troubleshoot the participant’s problems in adhering to prescribed interventions (TIB, relaxation). Address sleep effort and sleep-related anxiety. Discuss relapse prevention. Answer questions and address concerns. Provide instruction on how to continue increasing TIB if desired total sleep time is not yet achieved.

#### Control Condition

We will compare the CBT-I group to a 28-week waitlist control group. Participants assigned to the waitlist control group do not engage in any study interventions while on the waitlist (during the primary assessment period) but complete study assessments at weeks 1-6, 12, and 28. After completion of all primary study time points (28-week follow-up), those assigned to the waitlist control group receive the same 4 telehealth CBT-I sessions delivered over 5 weeks as did the CBT-I group (secondary assessment period). The waitlist control group also completes additional questionnaires during their 4 treatment sessions, but the collected data will not be included in primary analyses.

#### Data Collection

This study is conducted through Stanford Zoom and Stanford RedCap. The Stanford RedCap platform is developed and operated by Stanford Medicine Research Information Technology team. The RedCap platform services at Stanford are subsidized by (1) Stanford School of Medicine Research Office and (2) the National Center for Research Resources and the National Center for Advancing Translational Sciences, National Institutes of Health, through grant UL1 TR001085. All self-reported data are collected through questionnaires built on Stanford RedCap. Data from clinical interviews are entered into RedCap during the interview and then reviewed by the interviewer after the session. Once a participant is deemed eligible by the study team, they are randomized and scheduled for a second Zoom meeting with study staff. At this second Zoom meeting, they are notified of their study arm assignment by a research coordinator and complete week 1 questionnaires. If they are assigned to the treatment group, they meet with the therapist after completing week 1 questionnaires. For each following treatment session (weeks 2-5), participants meet with a research coordinator before meeting with the therapist. At the posttreatment time point (week 6), participants are sent a survey link to complete online questionnaires. If the participant is assigned to the waitlist control group, the week 1 session ends after completing the questionnaires. Participants in the waitlist control group are sent an email with a link to weekly surveys for each subsequent weekly session (weeks 2-6). Once the participant completes the week 1 questionnaires, they are considered enrolled in the study.

#### Recruitment, Enrollment Criteria, Screening, and Retention

Participants are adults in the United States aged 18 years or older who experienced new sleep disturbances after the start of the COVID-19 pandemic. Participants were recruited nationally through online postings, newsletters, and social media. A subset of participants was recruited from an ongoing survey-based observational study about sleep and well-being during the COVID-19 pandemic. All participants met the inclusion and exclusion criteria outlined in Textbox 1.

Interested participants completed online prescreening questions (Table 2) assessing insomnia symptoms before and after the pandemic began (March 1, 2020), insomnia symptom duration, any unstable medications, and seizure history. Participants who met the prescreening criteria (Table 2) were invited to a video call with study staff to further assess eligibility. During this call, a research coordinator explained details of the study procedures and obtained informed consent. Consenting participants and research staff obtaining consent signed the informed consent form via a RedCap survey with e-signature capabilities. Participants were emailed a copy of the signed consent form. After informed consent was obtained, a trained member of the study staff then administered clinical interviews to assess eligibility. Before ending the call, a research coordinator sent the participant a link to complete remaining online screening questionnaires at their own pace. After the participants completed all online screening questionnaires and screening interviews (described in the Measures section), the study team collectively determined if the inclusion and exclusion criteria were met.

During the screening session, sleep disturbance was assessed using the Duke Structured Interview for Sleep Disorders (hereafter referred to as Duke) [41] and ISI. Participants were excluded from the study if their reported sleep disturbance duration on the Duke began prior to the start of the pandemic or if another sleep disorder was primarily responsible for their symptoms. Two versions of the ISI were administered at screening: one assessing symptoms during the week before the pandemic began (past) and one assessing symptoms in the past 2 weeks (current). Participants were deemed eligible for the study if past ISI score was <10 and current ISI score was ≥10, as a score of 10 was found to be optimal in detecting insomnia in a community sample [36].

Participants with current or past psychosis, bipolar disorder, or epilepsy were excluded from the study due to safety concerns. Manipulating sleep increases the risk of seizures [42], mania [43], and psychosis [44] in individuals with a history of these conditions. Further, the brief version of CBT-I utilized in this study has not been validated as a reliable therapy in these populations. We administered the Mini-International Neuropsychiatric Interview (MINI) [45] to screen for psychosis and bipolar disorder and took a basic medical history to screen for history of seizures.

Participants currently abusing substances or taking over-the-counter or prescribed medications for sleep were not permitted in the study. To participate, all other medications must have been stable for at least 3 weeks, and medical conditions must have been deemed stable for at least 3 months by the study clinicians. However, hypnotics and other medications or supplements used to treat sleep disturbance were not permitted to be used during participation in the study. We collected information about current medication use as part of a basic medical history and assessed substance abuse or dependence using the MINI.

For eligible participants, data collected at the screening time point (including surveys after the session) are used as baseline measures.

**Criteria for study participation.**

**Inclusion Criteria**

1. Age 18 years or older
2. Having access to the internet and an email address
3. Acute subjective complaint of sleep disturbance (Insomnia Severity Index [ISI] before the pandemic <10 and current ISI ≥10) that began after March 1, 2020 or the COVID-19 pandemic (as reported during interview)
4. Living in the United States
5. Literate and fluent in English
6. Willingness to participate in the study, sign the consent, and complete majority of questionnaires

**Exclusion Criteria**

1. Presence of suicidal ideation representing high risk as measured by Sheehan-Suicide Tracking Scale (S-STS)
2. Use of medication specifically prescribed for sleep disturbance and unwilling or unable to discontinue more than 1 week prior to baseline data collection
3. Current or lifetime history of bipolar disorder or psychosis
4. Current substance abuse or dependence
5. Not able to verbalize understanding of involvement in research and provide written, informed consent
6. Unstable pharmacotherapy for other mental health disorders (<3 weeks since beginning new medication)
7. Severe impediment to vision, hearing, or hand movement likely to interfere with the ability to complete assessments or are unable or unlikely to follow study protocols
8. Working rotating shift that overlaps with midnight

**Table 2:** Prescreening questions and responses indicating eligibility for a screening session.

Prescreening questions	Response criteria for screening session
Are you currently taking any prescribed or over-the-counter sleep medication?	No or willing to discontinue medication prior to enrollment
Have you started a new medication within the last four weeks?	No
How many months have you had trouble sleeping?	Duration indicates symptoms started after the start of the COVID-19 pandemic (March 1, 2020)
Do you have a personal history of epilepsy, convulsions, or seizures?	No
Current Insomnia Severity Index	Total score ≥10
Past Insomnia Severity Index	Total score <10

We employ several precautionary measures to reduce attrition and retain participants. First, at the beginning of the study, participants met with a member of the study team to discuss study procedures and answer questions. Participants are encouraged to ask questions throughout their involvement in the study. Participants were assigned an assessor who administers their clinical interviews at every time point, to encourage familiarity and build rapport with study staff. Participants voluntarily provided multiple different types of contact information (eg, email, phone numbers) for appointment reminders, and sessions are scheduled based on participants’ time preferences. Study staff is persistent in attempting to recontact and engage noncompliant participants. Lastly, study assessments and data collection were carefully designed to minimize barriers to participation. We carefully curated the RedCap database so that participants do not have to re-enter information previously provided, and we eliminated long, superfluous questionnaires. We also added automated features to reduce the need for technical knowledge to navigate the questionnaires.

#### Protection Against Risks

All enrolled participants’ depressive and anxiety symptoms are monitored by a trained clinical psychologist who reviews all adverse events and any significant changes in well-being. Unlike many research studies that recruit locally and conduct study visits in person, the present study enrolls participants across the nation who may suffer from severe depressive symptoms such as suicidal ideation. To address this unique challenge, we developed a robust distressed patient protocol adapted from the 2009 model developed by Draucker et al [46]. Our protocol includes several precautionary measures and multiple levels of assessment and risk classification. Each participant was asked to provide the phone number and email address of an emergency contact during the consenting process. At the start of the first video treatment session, the visit provider confirms the address of the participant’s physical location and has the participant’s local emergency contact personnel readily available at each session. Study staff are trained to recognize signs of distress and indications that the participant may be having thoughts of harm to self or others. If such signs present over the course of the study visit, study staff conduct a formal risk assessment (using the Sheehan-Suicidality Tracking Scale [S-STS] if suicidal ideation is present or a homicide risk assessment if homicidal ideation is present) and determine if the participant is at imminent risk to self or others.

Study staff follow detailed instructions on how to proceed after a determination is made. If imminent threat is not determined, study participants are encouraged to follow up with their own mental health providers and are given contact information for their local emergency room, the National Suicide Prevention Lifeline, and the study psychologist. If imminent risk is determined, a warm transfer is provided to the National Suicide Prevention Lifeline. The visit provider then contacts the study psychologist, and together, they determine if contact of additional parties, including the participant’s emergency contact or local sheriff’s department, is warranted. At each stage, the study psychologist and principal investigator are apprised of steps taken, appropriate documentation is completed, and the institutional review board (IRB) is notified of any adverse events.

In addition to the aforementioned risk mitigation protocol, additional risk management strategies are utilized that leverage built-in, automated systems in RedCap if risk is detected during online survey completion. In RedCap, we established branching logic that automatically classifies subjects as low, moderate, or high suicide risk and sends precomposed emails based on risk level. Participants flagged as low, moderate, or high risk are sent direct emails with study staff contact information, as well as the National Suicide Prevention Lifeline. Those flagged as high risk are also contacted directly by the study psychologist who conducts a full risk assessment via telephone to ensure participant safety to self and others. Emergency contacts or local sheriff’s departments are contacted if a participant is deemed to be high-risk and cannot be directly reached.

### Measures

#### Screening

During the screening session, sleep disturbance was assessed using the Duke [41] and ISI. The Duke is a structured clinical interview that screens for sleep disorders in accordance with criteria of both the DSM-IV and the International Classification of Sleep Disorders, 2nd Edition, (ICSD-2). It is composed of 4 modules that assess sleep disorder symptoms associated with insomnia, hypersomnia, circadian rhythm sleep disorders, and sleep disorders associated with parasomnias.

The ISI is a 7-item, self-report measure of insomnia severity. The items consist of severity of early, middle, or late insomnia; sleep dissatisfaction; interference with daytime functioning; perception of sleep problems by others; and distress caused by sleep difficulties. Items are scored from 0 to 4, with 0 indicating no problem and 4 indicating a very severe problem. Score ranges for insomnia are as follows: 0-7, absent; 8-14, subthreshold; 15-21, moderate; and 22-28, severe.

We administered the MINI [45] to screen for psychosis, bipolar disorder, and substance abuse or dependence. The MINI is a structured, diagnostic interview for DSM-5 psychiatric disorders that was administered by clinically trained study staff.

Suicidal ideation and behaviors are assessed using the self-report version of the S-STS [47]. The S-STS is a 15-item questionnaire assessing the risk of suicidality using a 5-point Likert scale ranging from 0 to 4, with 0 indicating no problem and 4 indicating a very severe problem. For this study, to minimize participant burden, we omitted item 15, which asks about suicide attempts, because high-risk participants would already be identified through the distressed patient protocol (described in previous sections).

#### Primary Outcomes

Primary outcomes of insomnia, well-being, and predictors of treatment response are collected at baseline (week 0), weeks 6, 12, 28, and 56. Primary outcomes of insomnia (ISI) and well-being were also collected at weeks 1-5. The primary time points are weeks 6, 12, and 28.

##### Insomnia

The change in clinically significant insomnia symptoms (meeting criteria for insomnia disorder diagnosis) and subjective ratings of current insomnia symptoms are primary measures of insomnia. Insomnia disorder diagnosis is assessed using the Duke insomnia disorder module. Subjective sleep complaints are assessed using ISI report of current symptoms over the past 2 weeks. The Duke insomnia disorder module is only collected at weeks 0, 12, 28, and 56.

##### Well-being

We measure change in depressive symptoms as primary outcomes of well-being. Depressive symptoms are evaluated using the Patient Health Questionnaire-9 (PHQ-9) [48]. The PHQ-9 is a self-administered, 9-item questionnaire that assesses each of the 9 DSM-IV depression criteria. The total score ranges from 1 to 27. Answers are given on a 4-point Likert scale ranging from 0 (Not at all) to 3 (Nearly every day), with higher scores reflecting increased severity of depressive symptoms.

##### Predictors of Treatment Response

Baseline levels of sleep reactivity and pandemic-related risk factors, including loneliness, perceived stress, screen time, social connection, and physical activity, are measured as predictors of long-term treatment outcomes.

Loneliness is measured using the University of California, Los Angeles (UCLA) Loneliness Scale [49]. This self-report measure assesses participant's subjective feelings of loneliness and social isolation. The UCLA Loneliness Scale asks participants to rate how often each of the 20 items is descriptive of them, rated from 1 (never) to 4 (often). The responses are for an overall score range of 20 to 80, with higher scores indicating greater degrees of reported loneliness.

Sleep reactivity is measured by the Ford Insomnia Response to Stress Test (FIRST) [50]. This 9-item self-report tool measures risk of participants experiencing situational insomnia due to common stressful conditions. A higher score on the FIRST indicates higher sleep reactivity. The FIRST has good reliability and validity and has demonstrated high internal consistency across multiple demographic groups in clinical and population-based samples [51].

Perceived stress is measured using the Perceived Stress Scale (PSS) [52]. The PSS is a widely used 14-item self-report questionnaire that assesses how stressful participants believe their lives to be. Items are generalized and measure the degree to which participants judge their lives to have been uncontrollable and unpredictable over the course of the previous month. Items are scored on a 5-point Likert scale, with total scores ranging from 0 to 56 and higher scores indicating higher levels of perceived stress.

Screen time is measured through modified self-report questions in the Coronavirus Health Impact Survey (CRISIS) [53]. Participants are asked to estimate the number of hours spent per day, over the course of the 2 most recent weeks, watching television or digital media (eg. Netflix, YouTube, web surfing) or using social media (eg. FaceTime, Facebook, Instagram, Snapchat, Twitter, TikTok).

Social connection is measured by the Social Network Index (SNI) [54]. The SNI is a 12-item questionnaire that assesses participation in different types of social relationships. The 12 types of relationships (eg, friend, children, spouse, religious group member) are scored by the number of network members with which they communicate at least every 2 weeks. A higher score on the SNI indicates a larger social network.

Physical activity is evaluated using the International Physical Activity Questionnaire (IPAQ) [55]. The IPAQ assesses the time spent on an individual's physical activity across 5 life domains over the previous 7 days. The activity domains consist of physical activity related to work, transportation, housework and caring for family, and recreation and sports, as well as the amount of time spent sitting each day. Minutes of sitting and walking, as well as moderate-intensity (walking not included) and vigorous-intensity activities, were calculated for each domain and for the entire past 7 days. The IPAQ has high reliability and validity and has been widely used to measure comparable estimates of physical activity in large populations. A higher score on the IPAQ indicates an increased physical activity.

#### Secondary Outcomes

All secondary outcomes of insomnia and well-being are collected at weeks 0, 12, 28, and 56. Secondary measures of insomnia and the Generalized Anxiety Disorder-7 (GAD-7) are also collected at weeks 1-6.

##### Insomnia

Changes in sleep onset latency (SOL), number of awakenings, wake after sleep onset (WASO), total sleep time (TST), and sleep efficiency (SE) over time are measured as secondary measures of insomnia symptoms using sleep diaries; 7 days of sleep diaries are collected at baseline and weeks 1-5, 12, 28, and 56. Sleep diaries collect information about sleep and rise times, time in and out of bed, number of middle-of-the-night awakenings, duration of these awakenings, sleep quality, nap frequency and duration, and caffeine and alcohol consumption. SOL is the time in minutes from “lights out” to sleep onset. WASO is the sum of the total number of minutes of wakefulness occurring after sleep onset and before final awakening (sleep offset). TST is the total time spent asleep, from the start of sleep onset to sleep offset, subtracting any periods of wakefulness. SE is calculated as TST divided by total time spent in bed, multiplied by 100.

##### Well-being

Secondary measures of well-being include measures of anxiety symptoms, suicidal ideation, and quality of life, as well as an additional measure of depressive symptoms.

Anxiety symptoms over time are assessed using the GAD-7 [56]. The GAD-7 is a widely used diagnostic self-report scale that assesses severity of anxiety symptomology. The GAD-7 is a 7-item, 4-point Likert scale ranging from 0 to 3 that measures severity of anxiety symptoms over the previous 2 weeks, with a total score ranging from 0 to 21. Higher scores indicate more severe anxiety symptomology.

Suicidal ideation and behaviors over time are measured by the S-STS [47]. For this outcome measure, 14 items (excluding item 15) are summed for an overall score ranging from 0 to 56. Again, higher scores indicate more severe difficulties.

Quality of life is assessed using the 36-Item Short Form Health Survey (SF-36) [57]. The SF-36 is a 36-item self-report survey to assess comprehensive quality-of-life measures. It consists of 8 subscales: vitality, physical functioning, bodily pain, general health perceptions, physical role functioning, emotional role functioning, and mental health. This survey is widely used and has been proven to be a reliable indicator of quality-of-life measures. The global score range is 0 to 100, with higher scores indicating better health conditions.

The Beck Anxiety Inventory (BAI) [58] is used as a measure of anxiety symptom change over time. The BAI is a 21-item self-report scale that assesses the severity of anxiety symptoms. Items are scored from 0 to 3. Higher scores indicate greater levels of severity, and the ranges for anxiety levels are as follows: 0-9, normal to minimal; 10-18, mild to moderate; 19-29, moderate to severe; and 30-63, severe. The BAI consists of 2 factors: somatic and cognitive.

The Beck Depression Inventory-II (BDI-II) [59,60] is used as a secondary measure of depressive symptom change over time. We will sum all items except one sleep item, and the average item score for the remaining 20 items will be multiplied by 21 (the original number of items) to create a modified depression scale that maintains the original range (ranges: 0-13, minimal; 14-19, mild; 20-28, moderate; and 29-63, severe). The BDI-II is a 21-item self-report scale with high validity and reliability that assesses the severity of depressive symptoms. The depressive items consist of sadness, pessimism, past failure, loss of pleasure, guilty feelings, punishment feelings, self-dislike, self-criticalness, suicidal thoughts or wishes, crying, agitation, loss of interest, indecision, worthlessness, loss of energy, changes in sleep pattern, irritability, changes in appetite, concentration difficulty, tiredness or fatigue, and loss of interest in sex. Items are scored from 0 to 3, and higher scores indicate greater level of severity.

### Statistical Procedure and Data Analysis

Given the nature of this feasibility study, for all our analyses and interpretation, we will place a primary emphasis on estimation of effect sizes and confidence intervals rather than on testing statistical significance.

#### Primary Analyses

Statistical analyses aim 1 is to determine whether a brief, telehealth CBT-I reduces insomnia symptoms arising during the COVID-19 pandemic. All primary analyses will be performed using the intention-to-treat principle. We will test whether CBT-I is superior to a waitlist control in reducing insomnia symptoms by using a mixed effects linear model with autoregressive error structure using intention-to-treat analysis with outcomes at time points 6, 12, and 28. Insomnia severity as measured by the ISI will be entered as the dependent variable with randomization group (2 levels), time point (weeks 0, 6, 12, and 28), and group-by-time interaction included as fixed effects. The model will also include a random slope. Several hypotheses (1.1a-d) will be tested using the described mixed effects model.

Hypothesis 1.1a is that individuals assigned to the CBT-I group will experience an improved trajectory of insomnia symptoms during the waitlist-controlled 28-week period relative to those assigned to the waitlist control group. This hypothesis will be tested using a likelihood ratio test of the coefficients of the time and group-by-time interaction of the aforementioned mixed effects linear model.

Hypotheses 1.1b-1.1d are that individuals assigned to the CBT-I group during the waitlist-controlled period will have lower insomnia symptoms compared with those assigned to the waitlist-control group immediately posttreatment (hypothesis 1.1b; week 6) and at the short-term (hypothesis 1.1c; week 12) and long-term (hypothesis 1.1d; week 28) follow-ups. These hypotheses will be tested using the aforementioned mixed effects linear model with independent likelihood ratio tests using the treatment coefficient for the posttreatment (hypothesis 1.1b; Δ_1_ in Figure 2), short-term (hypothesis 1.1c; Δ_2_ in Figure 2), and long-term (hypothesis 1.1c; Δ_3_ in Figure 2) follow-up time points.

We will also test whether CBT-I is superior to the waitlist control in preventing an insomnia diagnosis following treatment by using multiple logistic regression analysis.

Hypotheses 1.2a and 1.2b are that individuals who were assigned to the CBT-I group will be less likely to have an insomnia diagnosis than those who were assigned to the waitlist control group at weeks 12 and 28. These hypotheses will be tested using 2 separate logistic regression models, one using the short-term (hypothesis 1.2a; 12 weeks) and one using long-term (hypothesis 1.2b; 28 weeks) follow-up insomnia diagnosis, as defined by the Duke, as the dependent variable, with treatment (CBT-I, waitlist) as the independent variable.

Statistical analyses aim 2 is to determine whether a brief, telehealth CBT-I improves well-being during the COVID-19 pandemic. We will test whether immediate CBT-I is superior to a waitlist control in improving depressive symptoms as the primary measure of well-being during the waitlist-controlled period (28-week duration) by using a mixed effects linear model with autoregressive error structure using intention-to-treat analysis. Depression severity, as assessed by the BDI-II, will be entered as the dependent variable with baseline covariates used for stratified randomization (ie, baseline insomnia severity and sex), group (immediate CBT-I or waitlist control), time point (weeks 0-6, 12, and 28), and group-by-time interaction included as fixed effects. The model will also include a random slope. Separate, secondary analyses will be conducted on the secondary measures relating to well-being described in the Secondary Outcomes section (eg, anxiety, suicidality, quality of life). Several hypotheses will be tested using this model.

Hypothesis 2.2a is that individuals assigned to the CBT-I group will have an improved trajectory of depressive symptoms across the 28 weeks relative to individuals assigned to the waitlist control group. This hypothesis will be tested using a likelihood ratio test of the coefficients of the time and group-by-time interaction terms of the aforementioned mixed effects linear model.

Hypotheses 2.2b-2.2d are that individuals assigned to the CBT-I group will have lower depressive symptoms compared who those who were assigned to the waitlist control group immediately posttreatment (hypothesis 2.2b; week 6) and at the short-term (hypothesis 2.2c; week 12) and long-term (hypothesis 2.2d; week 28) follow-ups. These hypotheses will be tested using the aforementioned mixed effects linear model with independent likelihood ratio tests using the treatment coefficient for the posttreatment (hypothesis 1.2b; week 6), short-term (hypothesis 1.2c; week 12), and long-term (hypothesis 1.2c; week 28) follow-up time points.

**Figure 3:**
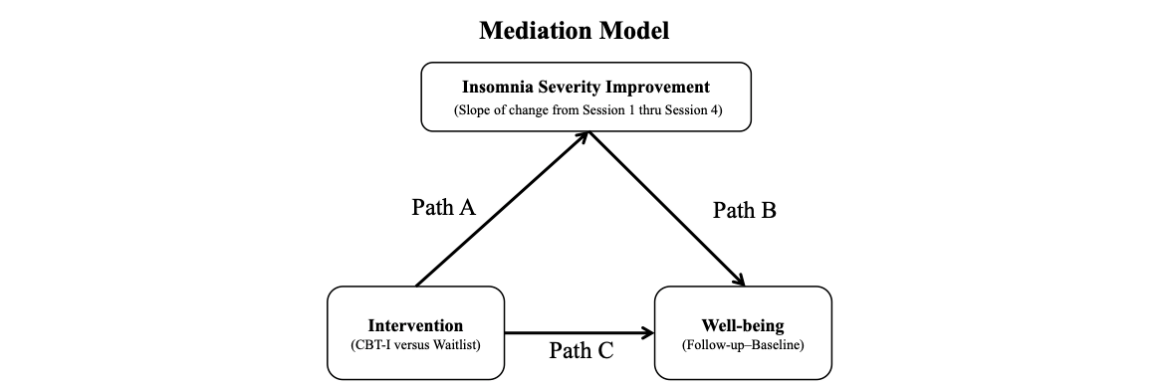
Mediation Model of Insomnia Severity Improvements mediating the change in well-being associated with the intervention.

Hypothesis 2.3 is that improvement in insomnia symptom severity (measured by the ISI) will mediate subsequent improvement in well-being from baseline (week 0) to posttreatment (week 6), short-term follow-up (week 12), and long-term follow-up (week 28). Using the approach described by Kraemer at al [61] and Manber et al [34], linear regression analyses will be used to determine whether change in the ISI score from Session 1 (week 1) to Session 4 (week 5) of treatment mediates the subsequent depressive symptom improvement (primary outcome measure) after the completion of treatment (week 6). Change in ISI score at Session 4 will be estimated as the slope of the regression line of ISI scores from Session 1 (week 1) through the treatment phase to Session 4 (week 6) using all available data from each participant. The model will include group (CBT-I or waitlist control), change in ISI score from Session 1 to Session 4 (participant specific slopes), and their interaction with depression severity, as measured by the PHQ-9, as the dependent variables. Mediation will be tested using the MacArthur approach as described by Kraemer et al [62]. Specifically, a mediation effect requires a significant effect of the intervention on the mediator (Path A in Figure 3) and a significant association between the mediator and the outcome (Path B in Figure 3).

Statistical analyses aim 3 is to determine whether risk factors for insomnia that are aggravated during the COVID-19 pandemic predict worse insomnia and well-being outcomes at follow-up.

Hypothesis 3 is that high levels of social isolation, perceived stress, sleep reactivity, and screen time and low levels of physical activity caused by the COVID-19 pandemic will collectively predict a worse long-term outcome across both intervention arms. Two separate linear mixed models will be conducted for each outcome variable (insomnia severity and depressive symptoms). In each model, the outcome variable at 12 weeks and 28 weeks will be entered as the dependent variable with social isolation, perceived stress, sleep reactivity, screen time, and physical activity measures at baseline as well as experimental arm entered as predictors.

#### Secondary Analyses

Secondary analyses will be conducted. (1) Analytic methods described in the primary aims will be repeated but applied to secondary measures of sleep disturbance and well-being outcomes as described in the Secondary Outcomes section. (2) Analytic methods described in the primary aims will be repeated but applied to outcome measures collected at week 56. (3) Sparse unsupervised clustering and principal component analysis analyses will be used to identify cohesive factors of dysfunction in sleep complaints and patterns of mental health outcomes at baseline. Regression models will be used to quantify the relationships within and between sleep complaints and patterns of mental health outcomes. (4) Age and sex differences in treatment response and as moderators of relationships between sleep complaints, insomnia risk factors, and mental health outcomes will be explored.

### Ethics Approval and Trial Registration

The IRB of Stanford University (Stanford, CA) approved the study, which is performed following the rules of the seventh edition (2013) of the Declaration of Helsinki (IRB-55940). It received initial approval from the IRB on April 30, 2020.

## Results

### Trial Status and Timeline

The trial began recruitment on June 4, 2020, and the first participant was consented on June 10, 2020. As of October 2021, 794 subjects had completed prescreening, and 96 subjects had completed a Zoom screening session. A total of 49 participants were randomized to a study group (26 to the CBT-I group and 23 to the waitlist control group). Overall, 38 participants had completed the week 6 follow-up, 37 had completed the week 12 follow-up, 34 had completed the week 28 follow-up, and 15 had completed the week 56 follow-up. At the time of this writing, there were an additional 3 participants awaiting the 28-week follow-up and a total of 23 participants awaiting the week 56 follow-up (13 in CBT-I and 10 in waitlist control group). The study closed to enrollment of new subjects on June 17, 2021. As of the writing of this paper, due to the outstanding data collection of the remaining 28- and 56-week data, we had conducted interim analyses for the week 6 follow-up but had not tested the results of our primary hypotheses in full. We expect primary results of the study to be published in 2022.

## Discussion

Here, we outline the protocol of an innovative research project responding to the mental health crisis related to the COVID-19 pandemic. We aim to address several gaps in the literature by investigating the use of an early, brief, nonpharmacological insomnia intervention delivered via telehealth to treat insomnia symptoms arising during a stressful life event and prevent worsening insomnia or mental health outcomes. This study represents multiple levels of innovation. First, to the best of our knowledge, this is the only project clinically responding to the large-scale increase in sleep disturbance during the COVID-19 pandemic. Although many studies are documenting the robust changes in sleep during the pandemic, this project is the first to test an intervention and examine pandemic-related predictors of intervention response. Second, on a broader scale, this is one of only a few studies testing the prospective temporal relationship between sleep disturbance and well-being. Lastly, to our knowledge, this is the only study of early deployment of CBT-I to treat insomnia symptoms arising from a stressful global event.

This study was conceived and launched in response to the global pandemic of COVID-19, which, while rendering it novel, presents unique challenges. First, to address our scientific questions, we sought to recruit participants early in the course of their sleep disturbance, causing a significant urgency to launch the study. This time pressure led us to conduct this study with limited resources, which impacted our recruitment efforts. We were unable to offer compensation for participation. We relied heavily on recruiting participants through free online platforms, such as social media posts, online message boards, and electronic newsletters. Due to these recruitment concerns and the resulting decreased statistical power for the planned analysis, we view this as a feasibility study. Therefore, the planned analyses and publications resulting from this protocol will utilize effect size estimates, rather than statistical significance, to provide pilot and feasibility data to inform future hypotheses. A second potential limitation is the unequal attrition rate in the treatment versus the waitlist control group. Since participants are not financially compensated, the primary motivation of participation is meeting with a therapist on a weekly basis to address their sleep concerns. Thus, it is possible that individuals in the waitlist control group were more likely to decline to participate in follow-up time points due to loss of interest or spontaneous improvement in symptoms over time. Although this could potentially affect the ability to detect significant differences in between-group comparisons at the long-term follow-up time points due to inadequate power, our analytic approach using regression models has the advantage of increased efficiency and power over unadjusted analyses [63,64]. We will address this issue by assessing whether missingness is related to any observed covariates. If this is the case, an issue of greater concern is the potential bias in the estimate of the treatment effect if neither the missing completely at random (MCAR) nor the missing at random (MAR) assumption holds. Although mixed effects linear models lead to unbiased estimates of the treatment effect under the MCAR and MAR assumptions, estimates may be biased under nonignorable missingness. Further sensitivity analyses will be performed if this is the case.

Although the unique conditions created by the COVID-19 pandemic posed many novel challenges, it also raises the potential significance of this protocol and feasibility study: It is unlikely that we or another group will be able to replicate this study in the future. Therefore, although the sample will be smaller than nonpandemic trials, it will likely be the largest sample to report on a sleep intervention deployed during the acute stage of a global pandemic. Supporting our hypothesis, the findings of a recent, large-scale clinical trial indicate that treatment of insomnia with CBT-I has an overall benefit in the prevention of incidence and recurrence of major depression in older adults with insomnia disorder. However, this trial utilized an in-person intervention in subjects with chronic insomnia [65]. Our study remains important by extending these findings to address the public health need for an effective, remote intervention to treat insomnia early and potentially prevent negative well-being outcomes. This project will lay the groundwork for future studies investigating early deployment of CBT-I to treat insomnia precipitated by a stressful event and whether this prevents adverse mental health outcomes for which poor sleep is a risk factor. Further, our investigation of pandemic-related risk factors for insomnia could guide future hypotheses for studies conducted in nonpandemic conditions.

## Abbreviations

BAI: Beck Anxiety Inventory
BDI-II: Beck Depression Inventory-II
CBT-I: cognitive behavioral therapy for insomnia
CRISIS: Coronavirus Health Impact Survey
DSM-5: Diagnostic and Statistical Manual of Mental Disorders 5th Edition
Duke: Duke Structured Interview for Sleep Disorders
FIRST: Ford Insomnia Response to Stress Test
GAD-7: Generalized Anxiety Disorder-7
ICSD-2: International Classification of Sleep Disorders, 2nd Edition
IPAQ: International Physical Activity Questionnaire
IRB: institutional review board
ISI: Insomnia Severity Index
MAR: missing at random
MCAR: missing completely at random
MINI: Mini-International Neuropsychiatric Interview
PHQ-9: Patient Health Questionnaire-9
PSS: Perceived Stress Scale
SE: sleep efficiency
SF-36: 36-Item Short Form Health Survey
SNI: Social Network Index
SOL: sleep onset latency
S-STS: Sheehan Suicidality Tracking Scale
TST: total sleep time
UCLA: University of California, Los Angeles
WASO: wake after sleep onset

## References

[1] Marelli S, Castelnuovo A, Somma A, Castronovo V, Mombelli S, Bottoni D, Leitner C, Fossati A, Ferini-Strambi L (2021). Impact of COVID-19 lockdown on sleep quality in university students and administration staff. J Neurol.

[2] Li Y, Qin Q, Sun Q, Sanford LD, Vgontzas AN, Tang X (2020). Insomnia and psychological reactions during the COVID-19 outbreak in China. J Clin Sleep Med.

[3] Hyun S, Hahm HC, Wong GTF, Zhang E, Liu CH (2021). Psychological correlates of poor sleep quality among U.S. young adults during the COVID-19 pandemic. Sleep Med.

[4] Gualano MR, Lo Moro G, Voglino G, Bert F, Siliquini R (2020). Effects of Covid-19 lockdown on mental health and sleep disturbances in Italy. Int J Environ Res Public Health.

[5] Yu BY, Yeung W, Lam JC, Yuen SC, Lam SC, Chung VC, Chung K, Lee PH, Ho FY, Ho JY (2020). Prevalence of sleep disturbances during COVID-19 outbreak in an urban Chinese population: a cross-sectional study. Sleep Med.

[6] Kokou-Kpolou CK, Megalakaki O, Laimou D, Kousouri M (2020). Insomnia during COVID-19 pandemic and lockdown: Prevalence, severity, and associated risk factors in French population. Psychiatry Res.

[7] LeBlanc M, Mérette C, Savard J, Ivers H, Baillargeon L, Morin CM (2009). Incidence and risk factors of insomnia in a population-based sample. Sleep.

[8] Ohayon MM (2002). Epidemiology of insomnia: what we know and what we still need to learn. Sleep Med Rev.

[9] Lai J, Ma S, Wang Y, Cai Z, Hu J, Wei N, Wu J, Du H, Chen T, Li R, Tan H, Kang L, Yao L, Huang M, Wang H, Wang G, Liu Z, Hu S (2020). Factors associated with mental health outcomes among health care workers exposed to coronavirus disease 2019. JAMA Netw Open.

[10] Pinto J, van Zeller M, Amorim P, Pimentel A, Dantas P, Eusébio E, Neves A, Pipa J, Santa Clara E, Santiago T, Viana P, Drummond M (2020). Sleep quality in times of Covid-19 pandemic. Sleep Med.

[11] Stanton R, To QG, Khalesi S, Williams SL, Alley SJ, Thwaite TL, Fenning AS, Vandelanotte C (2020). Depression, anxiety and stress during COVID-19: associations with changes in physical activity, sleep, tobacco and alcohol use in Australian adults. Int J Environ Res Public Health.

[12] Zhang Y, Zhang H, Ma X, Di Q (2020). Mental health problems during the COVID-19 pandemics and the mitigation effects of exercise: a longitudinal study of college students in China. Int J Environ Res Public Health.

[13] Grossman ES, Hoffman YSG, Palgi Y, Shrira A (2021). COVID-19 related loneliness and sleep problems in older adults: Worries and resilience as potential moderators. Pers Individ Dif.

[14] Cellini N, Canale N, Mioni G, Costa S (2020). Changes in sleep pattern, sense of time and digital media use during COVID-19 lockdown in Italy. J Sleep Res.

[15] Hale L, Guan S (2015). Screen time and sleep among school-aged children and adolescents: a systematic literature review. Sleep Med Rev.

[16] Gubelmann C, Heinzer R, Haba-Rubio J, Vollenweider P, Marques-Vidal P (2018). Physical activity is associated with higher sleep efficiency in the general population: the CoLaus study. Sleep.

[17] Deng J, Zhou F, Hou W, Silver Z, Wong CY, Chang O, Huang E, Zuo QK (2021). The prevalence of depression, anxiety, and sleep disturbances in COVID-19 patients: a meta-analysis. Ann N Y Acad Sci.

[18] Wang C, Pan R, Wan X, Tan Y, Xu L, Ho CS, Ho RC (2020). Immediate psychological responses and associated factors during the initial stage of the 2019 Coronavirus Disease (COVID-19) Epidemic among the general population in China. Int J Environ Res Public Health.

[19] Qiu J, Shen B, Zhao M, Wang Z, Xie B, Xu Y (2020). A nationwide survey of psychological distress among Chinese people in the COVID-19 epidemic: implications and policy recommendations. Gen Psychiatr.

[20] Huckins JF, daSilva AW, Wang W, Hedlund E, Rogers C, Nepal SK, Wu J, Obuchi M, Murphy EI, Meyer ML, Wagner DD, Holtzheimer PE, Campbell AT (2020). Mental health and behavior of college students during the early phases of the COVID-19 pandemic: longitudinal smartphone and ecological momentary assessment study. J Med Internet Res.

[21] Fernández-Abascal EG, Martín-Díaz MD (2021). Longitudinal study on affect, psychological well-being, depression, mental and physical health, prior to and during the COVID-19 pandemic in Spain. Pers Individ Dif.

[22] Romero-Gonzalez B, Puertas-Gonzalez JA, Mariño-Narvaez C, Peralta-Ramirez MI (2021). [Confinement variables by COVID-19 predictors of anxious and depressive symptoms in pregnant women]. Med Clin (Barc).

[23] Hertenstein E, Feige B, Gmeiner T, Kienzler C, Spiegelhalder K, Johann A, Jansson-Fröjmark M, Palagini L, Rücker G, Riemann D, Baglioni C (2019). Insomnia as a predictor of mental disorders: A systematic review and meta-analysis. Sleep Med Rev.

[24] Qaseem A, Kansagara D, Forciea MA, Cooke M, Denberg TD, Clinical Guidelines Committee of the American College of Physicians (2016). Management of Chronic Insomnia Disorder in Adults: A Clinical Practice Guideline From the American College of Physicians. Ann Intern Med.

[25] Trauer JM, Qian MY, Doyle JS, Rajaratnam SMW, Cunnington D (2015). Cognitive behavioral therapy for chronic insomnia: a systematic review and meta-analysis. Ann Intern Med.

[26] Koffel EA, Koffel JB, Gehrman PR (2015). A meta-analysis of group cognitive behavioral therapy for insomnia. Sleep Med Rev.

[27] Arnedt JT, Conroy DA, Mooney A, Furgal A, Sen A, Eisenberg D (2021). Telemedicine versus face-to-face delivery of cognitive behavioral therapy for insomnia: a randomized controlled noninferiority trial. Sleep.

[28] Altena E, Baglioni C, Espie CA, Ellis J, Gavriloff D, Holzinger B, Schlarb A, Frase L, Jernelöv S, Riemann D (2020). Dealing with sleep problems during home confinement due to the COVID-19 outbreak: Practical recommendations from a task force of the European CBT-I Academy. J Sleep Res.

[29] Gebara MA, Siripong N, DiNapoli EA, Maree RD, Germain A, Reynolds CF, Kasckow JW, Weiss PM, Karp JF (2018). Effect of insomnia treatments on depression: A systematic review and meta-analysis. Depress Anxiety.

[30] Bélanger L, Harvey AG, Fortier-Brochu E, Beaulieu-Bonneau S, Eidelman P, Talbot L, Ivers H, Hein K, Lamy M, Soehner AM, Mérette C, Morin CM (2016). Impact of comorbid anxiety and depressive disorders on treatment response to cognitive behavior therapy for insomnia. J Consult Clin Psychol.

[31] Luik AI, Kyle SD, Espie CA (2017). Digital cognitive behavioral therapy (dCBT) for insomnia: a state-of-the-science review. Curr Sleep Med Rep.

[32] Trockel M, Karlin BE, Taylor CB, Brown GK, Manber R (2015). Effects of cognitive behavioral therapy for insomnia on suicidal ideation in veterans. Sleep.

[33] Scott A, Webb T, Martyn-St James M, Rowse G, Weich S (2021). Improving sleep quality leads to better mental health: A meta-analysis of randomised controlled trials. Sleep Med Rev.

[34] Manber R, Buysse DJ, Edinger J, Krystal A, Luther JF, Wisniewski SR, Trockel M, Kraemer HC, Thase ME (2016). Efficacy of cognitive-behavioral therapy for insomnia combined with antidepressant pharmacotherapy in patients with comorbid depression and insomnia: a randomized controlled trial. J Clin Psychiatry.

[35] Morin CM (1993). Insomnia: Psychological assessment and management.

[36] Morin CM, Belleville G, Bélanger L, Ivers H (2011). The Insomnia Severity Index: psychometric indicators to detect insomnia cases and evaluate treatment response. Sleep.

[37] Borbely AA (1992). Concepts and models of sleep regulation: a preface. Journal of Sleep Research.

[38] Spielman AJ, Saskin P, Thorpy MJ (1987). Treatment of chronic insomnia by restriction of time in bed. Sleep.

[39] Bootzin RR, Epstein D, Wood JM, Hauri PJ (1991). Stimulus Control Instructions. Case Studies in Insomnia. Critical Issues in Psychiatry (An Educational Series for Residents and Clinicians).

[40] Edinger J Treatment Manual Cognitive-Behavioral Insomnia Therapy.

[41] Edinger J, Kirby A, Lineberger M, Loiselle M, Wohlgemuth W, Means M (2009). The Duke Structured Interview Schedule for DSM-IV-TR and International Classification of Sleep Disorders. ICSD-2 Sleep Disord Diagnoses.

[42] Bazil CW (2019). Seizure modulation by sleep and sleep state. Brain Res.

[43] Kaplan KA, Harvey AG (2013). Behavioral treatment of insomnia in bipolar disorder. Am J Psychiatry.

[44] Reeve S, Sheaves B, Freeman D (2015). The role of sleep dysfunction in the occurrence of delusions and hallucinations: A systematic review. Clin Psychol Rev.

[45] Sheehan DV, Lecrubier Y, Sheehan KH, Amorim P, Janavs J, Weiller E, Hergueta T, Baker R, Dunbar GC (1998). The Mini-International Neuropsychiatric Interview (M.I.N.I.): the development and validation of a structured diagnostic psychiatric interview for DSM-IV and ICD-10. J Clin Psychiatry.

[46] Draucker CB, Martsolf DS, Poole C (2009). Developing distress protocols for research on sensitive topics. Arch Psychiatr Nurs.

[47] Sheehan DV, Giddens JM, Sheehan IS (2014). Status update on the Sheehan-Suicidality Tracking Scale (S-STS) 2014. Innov Clin Neurosci.

[48] Kroenke K, Spitzer RL, Williams JB (2001). The PHQ-9: validity of a brief depression severity measure. J Gen Intern Med.

[49] Russell DW (1996). UCLA Loneliness Scale (Version 3): reliability, validity, and factor structure. J Pers Assess.

[50] Kalmbach DA, Pillai V, Arnedt JT, Drake CL (2016). Identifying at-risk individuals for insomnia using the Ford Insomnia Response to Stress Test. Sleep.

[51] Jarrin DC, Chen IY, Ivers H, Drake CL, Morin CM (2016). Temporal stability of the Ford Insomnia Response to Stress Test (FIRST). J Clin Sleep Med.

[52] Cohen S, Kamarck T, Mermelstein R (1983). A global measure of perceived stress. Journal of Health and Social Behavior.

[53] Nikolaidis A, Paksarian D, Alexander L, Derosa J, Dunn J, Nielson DM, Droney I, Kang M, Douka I, Bromet E, Milham M, Stringaris A, Merikangas KR (2021). The Coronavirus Health and Impact Survey (CRISIS) reveals reproducible correlates of pandemic-related mood states across the Atlantic. Sci Rep.

[54] Cohen S, Doyle WJ, Skoner DP, Rabin BS, Gwaltney JM (1997). Social ties and susceptibility to the common cold. JAMA.

[55] Craig CL, Marshall AL, Sjöström M, Bauman AE, Booth ML, Ainsworth BE, Pratt M, Ekelund U, Yngve A, Sallis JF, Oja P (2003). International physical activity questionnaire: 12-country reliability and validity. Med Sci Sports Exerc.

[56] Spitzer RL, Kroenke K, Williams JBW, Löwe B (2006). A brief measure for assessing generalized anxiety disorder: the GAD-7. Arch Intern Med.

[57] Hays RD, Sherbourne CD, Mazel RM (1993). The RAND 36-Item Health Survey 1.0. Health Econ.

[58] Beck AT, Epstein N, Brown G, Steer RA (1988). An inventory for measuring clinical anxiety: Psychometric properties. Journal of Consulting and Clinical Psychology.

[59] Beck AT, Steer RA, Ball R, Ranieri W (1996). Comparison of Beck Depression Inventories -IA and -II in psychiatric outpatients. J Pers Assess.

[60] Beck AT, Steer RA, Brown GK (1996). Manual for the Beck Depression Inventory-II.

[61] Kraemer HC, Wilson GT, Fairburn CG, Agras WS (2002). Mediators and moderators of treatment effects in randomized clinical trials. Arch Gen Psychiatry.

[62] Kraemer HC, Kiernan M, Essex M, Kupfer DJ (2008). Health Psychol.

[63] Moore KL, Neugebauer R, Valappil T, Laan MJ (2011). Robust extraction of covariate information to improve estimation efficiency in randomized trials. Stat Med.

[64] Benkeser D, Díaz I, Luedtke A, Segal J, Scharfstein D, Rosenblum M (2021). Improving precision and power in randomized trials for COVID-19 treatments using covariate adjustment, for binary, ordinal, and time-to-event outcomes. Biometrics.

[65] Irwin MR, Carrillo C, Sadeghi N, Bjurstrom MF, Breen EC, Olmstead R (2022). Prevention of incident and recurrent major depression in older adults with insomnia: a randomized clinical trial. JAMA Psychiatry.

